# Antibiotic utilisation and resistance over the first decade of nationally funded antimicrobial stewardship programmes in Singapore acute-care hospitals

**DOI:** 10.1186/s13756-023-01289-x

**Published:** 2023-08-23

**Authors:** Tat Ming Ng, Li Wei Ang, Shi Thong Heng, Andrea Lay-Hoon Kwa, Jia En Wu, Xue Fen Valerie Seah, Siok Ying Lee, Jonathan Seah, Robin Choo, Poh Lian Lim, Koh Cheng Thoon, Maciej Piotr Chlebicki, Jyoti Somani, Tau Hong Lee, David C. Lye

**Affiliations:** 1 Tan Tock Seng Hospital, Singapore, Singapore; 2 National Centre for Infectious Diseases, Singapore, Singapore; 3 Ministry of Health, Singapore, Singapore; 4 Singapore General Hospital, Singapore, Singapore; 5 National University Hospital, Singapore, Singapore; 6 KK Women’s and Children’s Hospital, Singapore, Singapore; 7 Khoo Teck Puat Hospital, Singapore, Singapore; 8 Changi General Hospital, Singapore, Singapore; 9 Ng Teng Fong General Hospital, Singapore, Singapore; 10 Duke- National University of Singapore Medical School, Singapore, Singapore; 11 Yong Loo Lin School of Medicine, Singapore, Singapore; 12 Lee Kong Chian School of Medicine, Singapore, Singapore

**Keywords:** Antimicrobial stewardship, Antimicrobial resistance, Decade, Singapore, National, Hospital

## Abstract

**Objective:**

The aim of this study was to describe the time series of broad-spectrum antibiotic utilisation and incidence of antibiotic-resistant organisms during the implementation of antimicrobial stewardship programmes (ASP) in Singapore.

**Methods:**

An observational study was conducted using data from 2011 to 2020 in seven acute-care public hospitals. We applied joinpoint regressions to investigate changes in antibiotic utilisation rate and incidence density of antibiotic-resistant organisms.

**Results:**

Across the seven hospitals, quarterly broad-spectrum antibiotic utilisation rate remained stable. Half-yearly incidence density of antibiotic-resistant organisms with two joinpoints at first half (H1) of 2012 and second half (H2) of 2014 decreased significantly in the second and third period with a half-yearly percentage change (HPC) of -2.9% and − 0.5%, respectively. Across the five hospitals with complete data, half-yearly broad-spectrum antibiotic utilisation rate with one joinpoint decreased significantly from H1 of 2011 to H2 of 2018 (HPC − 4.0%) and H2 of 2018 to H2 2020 (HPC − 0.5%). Incidence density of antibiotic-resistant organisms decreased significantly in the two joinpoint periods from H1 of 2012 to H2 of 2014 (HPC − 2.7%) and H2 of 2014 to H2 of 2020 (HPC − 1.0%). Ceftriaxone with one joinpoint decreased significantly from H1 of 2011 to H1 of 2014 (HPC − 6.0%) and H1 of 2014 to H2 of 2020 (HPC − 1.8%) and ceftriaxone-resistant *E. coli* and *K. pneumoniae* decreased significantly in later periods, from H2 of 2016 to H2 of 2020 (HPC − 2.5%) and H1 of 2012 to H2 of 2015 (HPC − 4.6%) respectively. Anti-pseudomonal antibiotics with one joinpoint decreased significantly from H1 of 2011 to H2 of 2014 (HPC − 4.5%) and H2 of 2014 to H2 of 2020 (HPC − 0.8%) and that of quinolones with one joinpoint at H1 of 2015 decreased significantly in the first period. C. *difficile* with one joinpoint increased significantly from H1 of 2011 to H1 of 2015 (HPC 3.9%) and decreased significantly from H1 of 2015 to H2 of 2020 (HPC − 4.9%).

**Conclusions:**

In the five hospitals with complete data, decrease in broad-spectrum antibiotic utilisation rate was followed by decrease in incidence density of antibiotic-resistant organisms. ASP should continue to be nationally funded as a key measure to combat antimicrobial resistance in acute care hospitals.

**Supplementary Information:**

The online version contains supplementary material available at 10.1186/s13756-023-01289-x.

## Background

Antimicrobial resistance (AMR) can be described as a tragedy of the commons that requires participation from a wide range of stakeholders and multiple levels of governance [1]. Globally, antimicrobial stewardship programme (ASP) has become an integral feature in most National Action Plans (NAPs) for AMR policies [2]. In 2011, Singapore implemented ASP in all its public acute care hospitals which comprised 83% (9762 of 11,704) of all acute-care hospital beds with plans to extend ASP to private hospitals, community healthcare facilities and the veterinary sector eventually [2, 3]. The Ministry of Health, Singapore provided SGD20 million to fund ASP in its public acute care hospitals, establishing multi-disciplinary teams comprising infectious disease physicians, microbiologists, pharmacists and data analysts [4]. Guidelines for the training and practice of ASP were subsequently developed [5]. To enable accountability, monitoring and evaluation, each hospital submits data on antibiotic utilisation and resistance, and intervention acceptance rates regularly.

In the ensuing years, studies from various public hospitals demonstrated that ASP improved the appropriateness of antibiotic prescribing and reduced the duration of antibiotic use without compromising patient safety [6–13]. Reductions in mortality, re-admissions and cost-savings were demonstrated [11–13]. As Singapore has reached a milestone in establishing ASP in all public acute care hospitals over 10 years, it is timely to conduct a review of broad-spectrum antibiotic utilisation and incidence density of antibiotic-resistant organisms to inform future national AMR policies. This study aimed to describe the time series of these two indicators over the first decade of public funding for ASP in seven acute care hospitals.

## Methods

### Study design

A 10-year observational study was conducted on broad-spectrum antibiotic utilisation and resistance data collected from seven public acute care hospitals in Singapore from 1 to 2011 to 31 December 2020. These acute care hospitals have different casemix and specialist services, ranging from a women’s and children’s hospital to hospitals with burns units, oncology and transplant services. In each hospital, antibiotic utilisation data was extracted from its pharmacy dispensing database and antibiotic resistance data extracted from its electronic laboratory reporting system. Broad-spectrum antibiotic utilisation data was submitted to the secretariat of the National Antimicrobial Resistance Control Committee (NARCC) on a quarterly basis and antibiotic resistance data on a half-yearly basis. In this study, we reported the initial list of antibiotics under NARCC surveillance and additional antibiotics added subsequently were excluded from our analysis. The broad-spectrum antibiotics included in the analysis were ceftazidime, ceftriaxone, cefepime, doripenem, ertapenem, imipenem, meropenem, intravenous (IV) amoxicillin-clavulanate, piperacillin-tazobactam, IV and oral (PO) ciprofloxacin, IV and PO levofloxacin, IV and PO moxifloxacin, colistin, polymyxin B, tigecycline, IV and PO linezolid, daptomycin and vancomycin.

With national ASP funding from 2011, the multi-disciplinary ASP teams of each hospital developed and disseminated antibiotic guidelines for the empiric treatment of common infections and surgical antibiotic prophylaxis. These were based on international guidelines and the antibiogram of each hospital. Prospective review and feedback (PRF) of inpatient antibiotic use targeting mainly carbapenems and piperacillin-tazobactam was conducted by each hospital based on these guidelines. Depending on the specific needs of each hospital, PRF was also conducted for cefepime, vancomycin, IV and PO ciprofloxacin and IV levofloxacin. A computerised decision support system was used in one hospital in 2011 and this increased to a total of four hospitals by the end of 2015 [14]. From 2015, all hospitals conducted activities planned in relation to the World Antimicrobial Awareness Week to raise awareness of appropriate antibiotic use. Educational activities such as in-house lectures, email messages and posters were shared with healthcare professionals.

Antibiotic utilisation was measured in defined daily doses (DDDs) per 1,000 inpatient-days while the incidence density of antibiotic-resistant organisms was reported as the number of clinical isolates per 1,000 inpatient-days. Isolates of the same organism from the same patient within a six-month period were counted only once. Multi-drug resistant (MDR) *A*. *baumannii* was defined as concurrent resistance to imipenem or meropenem, ciprofloxacin and amikacin. The World Health Organization (WHO) anatomical therapeutic chemical defined daily dose (ATC/DDD) index was used. The DDDs of meropenem, cefepime, colistin and IV ciprofloxacin were updated from 2 to 3 g, 2 to 4 g, 3 to 9 million units, and 0.5 to 0.8 g respectively in 2019 [15]. Antibiotics with similar DDDs for their IV and PO forms were calculated together (e.g., levofloxacin and linezolid). Two hospitals were unable to report complete data for PO ciprofloxacin, IV and PO levofloxacin and IV and PO moxifloxacin due to system limitations from 2011 to 2018. A separate analysis excluding the two hospitals with incomplete data was conducted to compare the trends with that of the analysis for the seven hospitals. Although the specific types of ASP activities were not specified in the data submitted by the hospitals, they were reported in the literature [6–13]. We defined acceptance as the primary doctor’s implementation of one or more of the ASP team’s recommendations upon receiving prospective review and feedback.

### Statistical analysis

We did not use interrupted time series analysis for this study as there were no pre-implementation data for comparison and the ASP activities of each hospital were not implemented at the same time. Hence, we used joinpoint regression models to identify the time points at which significant changes in rates of antibiotic utilisation and incidence density of antibiotic-resistant organisms occurred during the study period, which are also known as change points (“joinpoints”). We allowed up to five joinpoints for model fitting using the grid search method. A series of Monte Carlo permutation tests were used to select the optimal number of joinpoints, with Bonferroni correction for multiple testing.

Once the optimal joinpoints were identified, the time series was divided into distinct segments between each pair of consecutive joinpoints. Using a natural log-linear model within each segment, the size of the change was estimated as a constant percentage change over time, which was quarterly percentage change (QPC) for utilisation rate of antibiotics and half-yearly percentage change (HPC) for incidence density of antibiotic-resistant organisms. Assuming constant variance and correlated errors, we conducted a weighted least squares analysis in which the first-order autocorrelation parameter was estimated from the data [16]. The average QPC (AQPC) was calculated as a weighted average of the estimated QPC in each segment with segment lengths as weights, and likewise for average HPC (AHPC) [17].

All *p* values reported were 2-sided and statistical significance was taken as *p* < 0.05. The joinpoint regression analyses were performed using the Joinpoint Trend Analysis software from the Surveillance Research Programme of the National Cancer Institute Version 4.9.1.0 (Statistical Research and Applications Branch, National Cancer Institute, US) [18]. The figures were generated using R version 4.1.3 (R Foundation for Statistical Computing, Vienna, Austria).

## Results

The total number of inpatient-days from 2011 to 2020 for the seven acute care hospitals was 22,073,764. The two hospitals with incomplete data contributed 3,401,994 (15%) of the total inpatient-days. During this period, 87,010 ASP interventions were made across the seven hospitals with 65,551 (75.3%) accepted. Acceptance rate increased from 70.4% in first half (H1) of 2011 to 82.0% in second half (H2) of 2020; AHPC was 0.9% (95% confidence interval [CI], 0.3- to 1.4%). There were four joinpoints identified with significant increases in the third period (H2 of 2013, HPC of 1.9%, 95% CI 0.8- to 3.0%) and fourth period (H2 of 2015, HPC of 1.0%, 95% CI 0.9- to 1.2%).

### Broad-spectrum antibiotic utilisation of seven acute care hospitals

Overall quarterly rate of broad-spectrum antibiotic utilisation, excluding PO ciprofloxacin, IV and PO levofloxacin, and IV and PO moxifloxacin, remained stable across the seven hospitals during the study period with AQPC of -0.1%, 95% CI -0.2- to 0.01% (Table S1 and Figure S1). In Q1 of 2011, ceftriaxone had the highest utilisation followed by IV amoxicillin-clavulanate and piperacillin-tazobactam, whereas in Q4 of 2020, the latter two exceeded ceftriaxone in utilisation (Table S1, Figure S2).

The decrease in utilisation third-generation cephalosporins was mainly driven by ceftriaxone while the decrease in utilisation of carbapenems was primarily attributed to imipenem. Three joinpoints were detected for IV ciprofloxacin, and the only significant change in trend was in the first period from Q1 of 2011 to Q3 of 2016 with a QPC of-2.0%. The utilisation rate of IV beta-lactam/beta-lactamase inhibitors showed an increasing trend. Utilisation rate of IV amoxicillin-clavulanate significantly increased with a AQPC of 1.5%, from 40.1 to 62.0 DDDs/1,000 inpatient-days. There was one joinpoint at Q1 of 2013 for piperacillin-tazobactam with two periods of significant increase with the increase steeper in the first period: QPC of 3.8% vs. 0.5%. There was a significant decrease in the last joinpoint period for colistin (Q3 of 2015 to Q4 of 2020) and the last two joinpoint periods for polymyxin B (Q2 of 2013 to Q3 of 2019 and Q3 of 2019 to Q4 of 2020). Utilisation of ceftazidime (Q3 of 2013 to Q4 of 2020), piperacillin-tazobactam (Q1 of 2013 to Q4 of 2020), IV and PO linezolid (Q2 of 2018 to Q4 of 2020), daptomycin (Q3 of 2014 to Q4 of 2020) and vancomycin (Q2 of 2017 to Q4 of 2020) significantly increased in their last joinpoint period (Table S1, Figure S2).

### Broad-spectrum antibiotic utilisation of five acute care hospitals

A separate analysis of five acute care hospitals was conducted after excluding the two hospitals with incomplete data. Overall broad-spectrum antibiotic utilisation across the five acute care hospitals declined from 714.6 DDDs/1000 inpatient-days in Q1 of 2011 to 509.6 in Q4 of 2020 with AQPC of -0.8%, 95% CI -1.3 to -0.4% (Table S2, Figure S3). In Q1 of 2011, PO ciprofloxacin had the highest utilisation rate followed by ceftriaxone, piperacillin-tazobactam, IV amoxicillin-clavulanate, and PO and IV levofloxacin. In Q4 of 2020, PO ciprofloxacin was still in top rank and PO and IV levofloxacin surpassed the others in second place (Table S2, Figure S4).

Utilisation rate of anti-pseudomonal antibiotics (cefepime, ceftazidime, ciprofloxacin, colistin, doripenem, imipenem, meropenem, levofloxacin, piperacillin-tazobactam and polymyxin B) decreased significantly in the second joinpoint period (Q4 of 2012 to Q4 of 2013) and third period (Q4 of 2013 to Q4 of 2020). The decrease in third-generation cephalosporins was mainly driven by ceftriaxone while the decrease in carbapenems (Q1 of 2011 to Q3 2018, QPC of -0.2%) was mainly driven by imipenem. The decrease in quinolones was mainly driven by PO ciprofloxacin (Table S2, Figure S3 and Figure S4).

Utilisation rate of IV beta-lactam/beta-lactamase inhibitors increased significantly in the first joinpoint period (Q1 of 2011 to Q2 of 2014) and third period (Q4 of 2016 to Q3 of 2018). IV amoxicillin-clavulanate increased significantly in the second (Q4 of 2012 to Q1 2014, QPC of 3.5%) and fourth joinpoint periods (Q4 of 2015 to Q3 2018, QPC of 2.4%). There was one joinpoint at Q1 of 2013 for piperacillin-tazobactam with two periods of significant increase with the increase steeper in the first period from Q1 of 2011 to Q1 of 2013: QPC of 3.7% vs. Q1 of 2013 to Q4 of 2020, QPC of 0.3%. Both colistin and polymyxin B decreased significantly in their last periods. Piperacillin-tazobactam, PO and IV linezolid, and daptomycin increased significantly during their last periods (Table S2, Figure S4).

### Incidence density of antibiotic-resistant organisms of seven acute care hospitals

Overall incidence density of antibiotic-resistant organisms decrease significantly in the second (H1 of 2012 to H2 of 2014, HPC − 2.9%) and third period (H2 of 2014 to H2 of 2020, -0.5%) respectively. After excluding methicillin-resistant *S. aureus* (MRSA), a significant decrease in antibiotic-resistant organisms was still observed in the second joinpoint period from H1 of 2012 to H2 of 2014. Ciprofloxacin-resistant *E. coli* had the highest half-yearly incidence density, followed by ceftriaxone-resistant *E. coli*, ciprofloxacin-resistant *K. pneumoniae* and ceftriaxone-resistant *K. pneumonia*e.

Incidence density of ceftriaxone-resistant *E. coli* and *K. pneumoniae* decreased significantly in the last periods. Ciprofloxacin-resistant *E. coli* had one joinpoint at H2 of 2018 and increased significantly in the two periods with the increase steeper in the second period; HPC of 0.5% vs. 3.8%, while ciprofloxacin-resistant *K. pneumonia*e had three joinpoints and decreased significantly in the second period and increased significantly in the fourth period; HPC of -5.5% vs. 3.5%. Imipenem or meropenem-resistant *E. coli* and *K. pneumoniae* increased significantly with AHPC of 4.0% and 3.3%, respectively (Table 1, Figure S5).

**Table 1:** Joinpoint regression analysis of incidence density of antibiotic resistant organisms (isolates per 1,000 inpatient-days) across seven public acute-care hospitals, first half of 2011 to second half of 2020

Antibiotic resistant organism	No of isolates//1000 inpatient-days		Total study period (H1 2011–H2 2020)	Trend 1		Trend 2		Trend 3		Trend 4	
	H1 of 2011	H2 of 2020	AHPC (%) (95% CI)	Half-years	HPC (%) (95% CI)	Half-years	HPC (%) (95% CI)	Half-years	HPC (%) (95% CI)	Half-years	HPC (%) (95% CI)
All	11.9	11.0	-0.5 (-1.3, 0.4)	H1 2011- H1 2012	6.0 (-1.6, 14.2)	H1 2012- H2 2014	-2.9** (-4.6, -1.2)	H2 2014- H2 2020	-0.5** (-0.8, -0.2)		
*E. coli* resistant to:											
ceftriaxone	2.0	2.1	0.4 (-0.8, 1.5)	H1 2011–H1 2012	7.7* (0.9, 14.8)	H1 2012–H1 2015	-0.5 (-1.9, 0.9)	H1 2015–H2 2016	4.0 (-2.5, 10.9)	H2 2016–H2 2020	-2.0*** (-2.6, -1.3)
ciprofloxacin	3.3	4.3	1.2** (0.4, 2.0)	H1 2011–H2 2018	0.5* (0.1, 1.0)	H2 2018–H2 2020	3.8* (0.2, 7.6)				
imipenem or meropenem	0.01	0.03	4.0*** (3.0, 4.9)								
*K. pneumoniae* resistant to:											
ceftriaxone	1.4	1.0	-1.6 (-3.4, 0.3)	H1 2011–H1 2012	4.6 (-10.4, 22.2)	H1 2012–H2 2014	-5.2* (-8.6, -1.6)	H2 2014–H1 2018	0.8 (-1.1, 2.7)	H1 2018–H2 2020	-3.6* (-6.0, -1.1)
ciprofloxacin	1.6	1.5	-0.2 (-1.9, 1.5)	H1 2011–H1 2012	5.6 (-8.5, 21.9)	H1 2012–H2 2014	-5.5** (-8.6, -2.3)	H2 2014–H1 2018	-0.6 (-2.3, 1.0)	H1 2018–H2 2020	3.5** (1.1, 5.9)
imipenem or meropenem	0.04	0.06	3.3* (0.7, 6.0)	H1 2011–H1 2016	8.4*** (4.5, 12.3)	H1 2016–H2 2020	-2.0 (-6.1, 2.2)				
*P. aeruginosa* resistant to imipenem or meropenem	0.3	0.3	-0.5 (-4.2, 3.4)	H1 2011–H1 2015	2.8* (0.6, 5.1)	H1 2015–H2 2016	-9.8 (-30.1, 16.3)	H2 2016–H2 2020	-0.04 (-2.2, 2.2)		
*A. baumannii* resistant to:											
imipenem or meropenem	0.5	0.1	-9.9*** (-12.4, -7.2)	H1 2011–H2 2012	8.1 (-10.4, 30.4)	H2 2012–H2 2020	-12.9*** (-13.9, -11.8)				
multiple drugs^1^	0.4	0.1	-10.1*** (-13.1, -7.0)	H1 2011–H2 2012	13.8 (-0.8, 41.2)	H2 2012–H2 2020	-14.0*** (-15.3, -12.6)				
Methicillin-resistant *S. aureus*	1.8	0.9	-3.3*** (-3.7, -2.9)	H1 2011–H2 2016	-4.0*** (-4.5, -3.6)	H2 2016–H2 2020	-2.4*** (-3.2, -1.6)				
Vancomycin-resistant enterococci	0.1	0.2	3.6 (-7.9, 16.4)	H1 2011–H1 2012	55.7 (-10.7, 171.2)	H1 2012–H2 2013	-20.9 (-53.9, 35.9)	H2 2013–H1 2015	22.2 (-28.8, 109.8)	H1 2015–H2 2020	-1.1 (-3.7, 1.6)
*C. difficile*	0.5	0.5	-0.9* (-1.7, -0.1)	H1 2011–H1 2015	3.8*** (2.2, 5.5)	H1 2015–H2 2020	-4.2*** (-5.1, -3.2)				
All except for methicillin-resistant *S. aureus*	10.1	10.1	-0.1 (-1.0, 0.9)	H1 2011– H1 2012	7.7 (-0.8, 16.8)	H1 2012– H2 2014	-2.7 (-4.6, -0.8)*	H2 2014– H2 2020	-0.2 (-0.5, 0.1)		

* *p* < 0.05; ** *p* < 0.01; ***p < 0.001

CI: confidence interval, H1: First half of the year, H2: Second half of the year, AHPC: average half-yearly percentage change

^1^ Multidrug-resistant *A. baumannii* was defined as concurrent resistance to imipenem or meropenem, ciprofloxacin and amikacin

Incidence density of imipenem or meropenem-resistant *P. aeruginosa* increased significantly in the first joinpoint period from H1 of 2011 to H1 of 2015 with HPC of 2.8%. Imipenem or meropenem-resistant *A. baumannii* and MDR *A. baumannii* had one joinpoint at H2 of 2012 and decreased significantly in the second period; HPC of -12.9% and − 14.0%. MRSA halved from 1.8 isolates/1,000 inpatient-days in H1 of 2011 to 0.9 in H2 of 2020. There was one joinpoint at H2 of 2016 for MRSA with two periods of significant decrease with the decline steeper in the first period: HPC of -4.0% vs. -2.4%. Vancomycin-resistant enterococci had three joinpoints but did not show significant changes in any of the four periods. *C. difficile* had one joinpoint at H1 of 2015 and increased significantly during the first period (HPC of 3.8%) and decreased significantly in the second period (HPC of -4.2%) (Table 1 and Figure S5).

### Comparison of broad-spectrum antibiotic utilisation to incidence density of antibiotic-resistant organisms

**Fig. 1:**
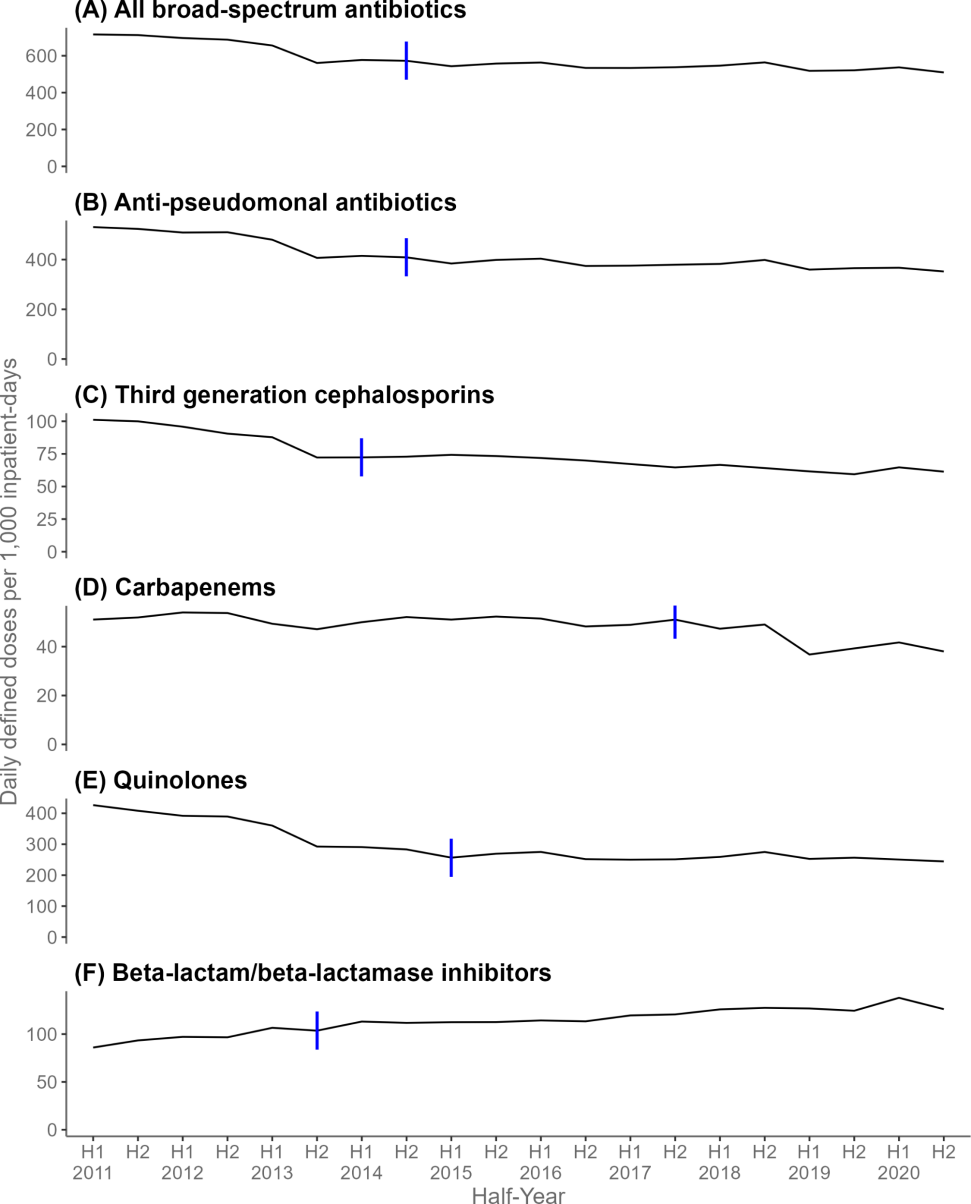
Trends in incidence density of antibiotic resistant organisms (isolates per 1,000 inpatient-days) across five public acute-care hospitals, first half of 2011 to second half of 2020. The | symbols denote the joinpoints identified using joinpoint regression analysis

**Fig. 2:**
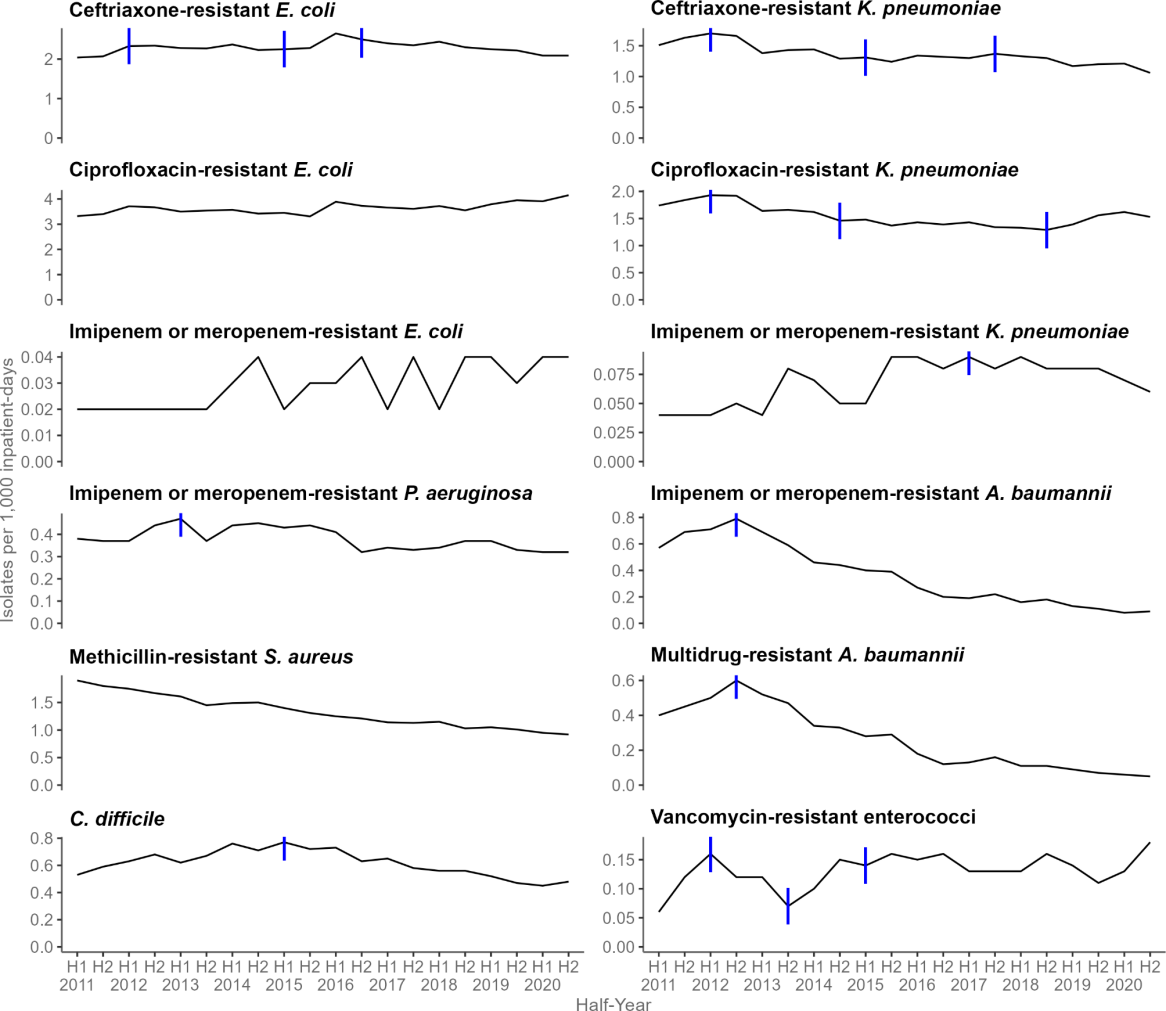
Trends in antibiotic utilisation rate (daily defined doses per 1,000 inpatient-days) across five public acute-care hospitals for (**A**) All broad-spectrum antibiotics, (**B**) Anti-pseudomonal antibiotics, (**C**) Third-generation cephalosporins, (**D**) Carbapenems, (**E**) Quinolones, (**F**) Beta-lactam/beta-lactamase inhibitors, first half of 2011 to second half of 2020. The | symbols denote the joinpoints identified using joinpoint regression analysis. Anti-pseudomonal antibiotics comprise cefepime, ceftazidime, ciprofloxacin, colistin, doripenem, imipenem, meropenem, levofloxacin, piperacillin-tazobactam and polymyxin B. Third generation cephalosporins comprise ceftazidime and ceftriaxone. Carbapenems comprise ertapenem, imipenem, meropenem and doripenem. Quinolones comprise ciprofloxacin, levofloxacin and moxifloxacin. Beta-lactam/beta-lactamase inhibitors comprise amoxicillin-clavulanate and piperacillin-tazobactam

We compared significant changes in broad-spectrum antibiotic utilisation rate with that of the incidence density of antibiotic-resistant organisms in half-yearly periods based on the five-hospital analyses (Tables 2 and 3; Figs. 1 and 2). There was one joinpoint detected at H2 of 2018 for the overall half-yearly antibiotic utilisation rate which decreased significantly in the two joinpoint periods (H1 of 2011 to H2 of 2018, HPC of -4.0% and H2 of 2018 to H2 of 2020, HPC of -0.5%). The overall incidence density of antibiotic-resistant organisms decreased significantly in the two joinpoint periods (H1 of 2012 to H2 of 2014, HPC of -2.7% and H2 of 2014 to H2 of 2020, HPC of -1.0%). After excluding MRSA, a significant decrease in antibiotic-resistant organisms was still observed in the joinpoint periods from H1 of 2012 to H2 of 2014 (HPC of -2.6%) and H2 of 2014 to H2 of 2020 (HPC of -0.7%).

**Table 2:** Joinpoint regression analysis of antibiotic utilisation rate (defined daily doses per 1,000 inpatient-days) across five public acute-care hospitals for selected antibiotics, H1 of 2011 to H2 of 2020

Antibiotic	DDDs/1,000 inpatient-days		Total study period (H1 2011–H2 2020)	Trend 1		Trend 2		Trend 3		Trend 4	
	H1 2011	H2 2020	AHPC (%), (95% CI)	Half-years	HPC (%) (95% CI)	Half-years	HPC (%) (95% CI)	Half-years	HPC (%), (95% CI)	Half-years	HPC (%), (95% CI)
**All**	715.6	509.5	-1.8*** (-2.3, -1.3)	H1 2011–H2 2018	-4.0*** (-5.2, -2.8)	H2 2018–H2 2020	-0.5* (-1.1, -0.01)				
**Anti-pseudomonal antibiotics** ^**1**^	530.6	352.1	-2.2*** (-2.7, -1.7)	H1 2011–H2 2014	-4.5*** (-5.8, -3.3)	H2 2014–H2 2020	-0.8** (-1.4, -0.3)				
**Third generation cephalosporins** ^**2**^	101.1	61.4	-2.9*** (-3.5, -2.2)	H1 2011–H1 2014	-5.6*** (-7.4, -3.8)	H1 2014–H2 2020	-1.6*** (-2.1, -1.0)				
Ceftazidime, IV (g)	8.3	8.3	-0.6* (-1.0, -0.1)								
Ceftriaxone, IV (g)	92.9	53.1	-3.1*** (-3.8, -2.5)	H1 2011–H1 2014	-6.0*** (-7.8, -4.3)	H1 2014–H2 2020	-1.8*** (-2.3, -1.2)				
**Carbapenems** ^**3**^	51.1	38.0	-1.8*** (-2.7, -0.8)	H1 2011–H2 2017	-0.4 (-1.2, 0.5)	H2 2017–H2 2020	-4.7** (-7.4, -1.9)				
Doripenem, IV (g)	0.41	0.12	-5.7 (-11.6, 0.6)	H1 2011–H1 2015	-19.4** (-29.2, -8.4)	H1 2015–H2 2020	5.8 (-2.1, 14.2)				
Ertapenem, IV (g)	11.43	9.22	-1.5*** (-1.9, -1.0)								
Imipenem, IV (g)	10.7	0.89	-13.5*** (-15.2, -11.8)	H1 2011–H1 2016	-18.8*** (-21.0, -16.5)	H1 2016–H2 2020	-7.3*** (-10.3, -4.3)				
Meropenem, IV (g)	28.5	27.8	-0.1 (-1.2, 1.0)	H1 2011–H1 2016	3.7*** (2.1, 5.3)	H1 2016–H1 2020	-4.1*** (-5.8, -2.4)				
**Quinolones** ^**4**^	426.4	244.6	-2.9*** (-3.6, -2.2)	H1 2011–H1 2015	-6.3*** (-7.5, -5.0)	H1 2015–H2 2020	-0.4 (-1.2, 0.4)				
Ciprofloxacin, IV (g)	8.6	3.1	-5.3 (-10.4, 0.1)	H1 2011–H2 2016	-3.8*** (-4.9, -2.7)	H2 2016–H1 2018	2.8 (-20.2, 32.4)	H1 2018–H2 2019	-19.1 (-37.2, 4.2)	H2 2019–H2 2020	-2.8 (-25.8, 27.5)
Ciprofloxacin, PO (g)	366.9	164.1	-4.2*** (-4.8, -3.5)	H1 2011–H2 2014	-7.3*** (-8.8, -5.8)	H2 2014–H2 2020	-2.3*** (-3.0, -1.6)				
Levofloxacin, IV and PO (g)	42.5	76.0	3.2** (1.3, 5.1)	H1 2011–H1 2017	0.2 (-1.7, 2.1)	H1 2017–H2 2020	8.6** (4.1, 13.3)				
Moxifloxacin, IV and PO (g)	8.4	1.4	-8.4*** (-11.2, -5.6)	H1 2011–H2 2015	-12.3*** (-16.7, -7.8)	H2 2015–H2 2020	-4.8* (-8.8, -0.6)				
**Beta-lactam/beta-lactamse inhibitors** ^**5**^	86.0	126.1	2.2*** (1.8, 2.5)	H1 2011– H2 2013	3.9*** (2.5, 5.4)	H2 2013–H2 2020	1.5*** (1.3, 1.8)				
Amoxicillin-clavulanate, IV (g)	41.8	62.9	2.6*** (2.3, 2.9)								
Piperacillin-tazobactam, IV (g)	44.2	63.5	1.9*** (1.3, 2.5)	H1 2011–H1 2015	6.9*** (4.0, 10.0)	H1 2015–H2 2020	0.6** (0.3, 0.9)				
**Others**											
Cefepime, IV (g)	16.0	7.9	-4.3 (-9.5, 1.2)	H1 2011–H2 2015	-2.4* (-4.2, -0.6)	H2 2015–H1 2018	3.7 (-3.5, 11.4)	H1 2018–H2 2019	-22.6 (-43.4, 5.8)	H2 2019–H2 2020	-1.1 (-29.5, 38.6)
Colistin, IV (million units)	0.66	0.1	-8.6* (-15.1, -1.6)	H1 2011–H1 2013	22.9 (-13.5, 74.4)	H1 2013–H2 2020	-15.5*** (-18.9, -12)				
Polymyxin B, IV (g)	1.8	0.4	-9.1*** (-13.3, -4.8)	H1 2011–H1 2013	14.4* (2.5, 27.6)	H1 2013–H2 2019	-9.9*** (-11.5, -8.2)	H2 2019–H2 2020	-39.3* (-60.5, -6.9)	-	-
Tigecycline, IV (g)	0.9	1.1	1.1 (-0.5, 2.8)								
Linezolid, IV and PO (g)	2.9	2.6	-0.3 (-3.4, 2.8)	H1 2011–H1 2018	-7.8*** (-9.9, -5.7)	H1 2018– H2 2020	24.1** (10.9, 38.8)				
Daptomycin, IV (g)	0.9	2.8	6.9*** (5.1, 8.7)								
Vancomycin IV (g)	27.8	24.6	-0.8 (-3.4, 1.8)	H1 2011–H1 2012	2.4 (-9.5, 15.9)	H1 2012– H4 2013	-7.7 (-18.3, 4.2)	H4 2013–H1 2015	4.2 (-7.7, 17.7)	H1 2015–H4 2020	-0.8* (-1.4, -0.2)

* *p* < 0.05; ** *p* < 0.01; ***p < 0.001

CI: confidence interval, DDDs: daily defined doses, Q: quarter, QPC: quarterly percentage change, AQPC: average quarterly percentage change, IV: intravenous, PO: oral

^1^Anti-pseudomonal antibiotics comprise cefepime, ceftazidime, ciprofloxacin, colistin, doripenem, imipenem, meropenem, levofloxacin, piperacillin-tazobactam and polymyxin B. ^2^Third generation cephalosporins comprise ceftazidime and ceftriaxone. ^3^Carbapenems comprise ertapenem, imipenem, meropenem and doripenem. ^4^Quinolones comprise ciprofloxacin, levofloxacin and moxifloxacin. ^5^Beta-lactam/beta-lactamase inhibitors comprise amoxicillin-clavulanate and piperacillin-tazobactam

**Table 3:** Joinpoint regression analysis of incidence density of antibiotic resistant organisms (isolates per 1,000 inpatient days) across five public acute-care hospitals, first half of 2011, second half of 2020

Antibiotic resistant organism	No of isolates//1,000 inpatient-days		Total study period (H1 2011–H2 2020)	Trend 1		Trend 2		Trend 3		Trend 4	
	H1 of 2011	H2 of 2020	AHPC (%) (95% CI)	Half-years	HPC (%) (95% CI)	Half-years	HPC (%) (95% CI)	Half-years	HPC (%) (95% CI)	Half-years	HPC (%) (95% CI)
All	12.5	11.0	-0.7 (-1.6, 0.2)	H1 2011– H1 2012	6.0 (-2.7, 15.4)	H1 2012– H2 2014	-2.7** (-4.5,-0.9)	H2 2014– H2 2020	-1.0*** (-1.3, -0.7)		
*E. coli* resistant to:											
ceftriaxone	2.0	2.1	0.2 (-1.2, 1.7)	H1 2011– H1 2012	8.0 (-1.0, 17.7)	H1 2012– H1 2015	-0.3 (-1.7, 1.0)	H1 2015– H2 2016	3.9 (-4.4, 12.8)	H2 2016– H2 2020	-2.5*** (-3.1, -1.8)
ciprofloxacin	3.3	4.2	0.7*** (0.3, 0.1)								
imipenem or meropenem	0.02	0.04	3.9*** (2.8, 4.9)								
*K. pneumoniae* resistant to:											
ceftriaxone	1.5	1.1	-1.7 (-3.7, 0.3)	H1 2011– H1 2012	4.4 (-11.9, 23.7)	H1 2012– H2 2015	-4.6** (-7.2, -1.9)	H2 2015– H2 2017	1.6 (-2.5, 5.8)	H2 2017– H2 2020	-3.3** (-5.3, -1.3)
ciprofloxacin	1.7	1.5	-0.3 (-2.1, 1.6)	H1 2011– H1 2012	5.9 (-9.0, 23.3)	H1 2012– H2 2014	-5.4** (-8.9, -1.9)	H2 2014– H2 2018	-1.3 (-2.7, 0.1)	H2 2018–H2 2020	5.5* (1.6, 9.5)
imipenem or meropenem	0.04	0.1	3.1* (0.5, 5.7)	H1 2011– H1 2017	7.5*** (4.7, 10.3)	H1 2017– H2 2020	-4.0 (-9.7, 2.0)				
*P. aeruginosa* resistant to imipenem or meropenem	0.4	0.3	-0.6 (-2.5, 1.2)	H1 2011– H1 2013	5.7 (-3.1, 15.3)	H1 2013– H2 2020	-2.3*** (-3.3, -1.2)				
*A. baumannii* resistant to:											
imipenem or meropenem	0.6	0.1	-9.9*** (-12.6, -7.2)	H1 2011– H2 2012	8.4 (-10.6, 31.4)	H2 2012– H2 2020	-13.0*** (-14.1, -12.0)				
multiple drugs^1^	0.4	0.1	-10.1*** (-13.1, -7.0)	H1 2011– H2 2012	13.8 (-8.5, 41.7)	H2 2012– H2 2020	-14*** (-15.4, -12.6)				
Methicillin-resistant *S. aureus*	1.9	0.9	-3.7*** (-3.8, -3.5)								
Vancomycin-resistant enterococci	0.1	0.2	3.6 (-7.8, 16.6)	H1 2011– H1 2012	56.7 (-10.4, 173.9)	H1 2012– H2 2013	-20.9 (-54.0, 36.0)	H2 2013– H1 2015	22.4 (-28.8, 110.6)	H1 2015– H2 2020	-1.1 (-3.7, 1.6)
*C. difficile*	0.5	0.5	-1.3*** (-1.9, -0.8)	H1 2011– H1 2015	3.9*** (2.6, 5.2)	H1 2015– H2 2020	-4.9*** (-5.6, -4.2)				
All except for methicillin-resistant *S. aureus*	10.6	10.1	-0.3 (-1.4, 0.7)	H1 2011– H1 2012	7.7 (-2.2, 18.6)	H1 2012– H2 2014	-2.6* (-4.5, -0.5)	H2 2014– H2 2020	-0.7*** (-1.0, -0.3)		

* *p* < 0.05; ** *p* < 0.01; ***p < 0.001

CI: confidence interval, H1: First half of the year, H2: Second half of the year, AHPC: average half-yearly percentage change

^1^Multidrug-resistant *A. baumannii* was defined as concurrent resistance to imipenem or meropenem, ciprofloxacin and amikacin

Utilisation rate of ceftriaxone decreased significantly with one joinpoint detected at H2 of 2014 in the two periods, whereas the incidence density of ceftriaxone-resistant *E. coli* and *K. pneumoniae* decreased significantly in later periods (Figure S6). The utilisation rate of carbapenems with one joinpoint at H2 of 2017 decreased significantly in the second period whereas incidence density of imipenem or meropenem-resistant *E. coli* increased throughout the study period and imipenem or meropenem-resistant *K. pneumoniae* with one joinpoint at H1 of 2017 increased significantly in the second period. Imipenem or meropenem resistant-*P. aeruginosa* and imipenem or meropenem resistant-*A. baumannii*, each with one joinpoint detected at H1 of 2013 and H2 of 2012 respectively, decreased significantly in the second period (Figure S7). The utilisation rate of anti-pseudomonal antibiotics with one joinpoint at H2 of 2014 decreased in both periods while that of quinolones with one joinpoint at H1 of 2015 decreased significantly in the first period. C. *difficile* with one joinpoint at H1 of 2015 significantly increased in incidence density in the first period but significantly decreased in the second period (Figs. 1 and 2).

## Discussion

Our study on the five hospitals with complete data showed that the half-yearly utilisation rate of broad-spectrum antibiotics decreased significantly from H1 of 2011 to H2 of 2018, while the first significant decrease in half-yearly incidence density of antibiotic-resistant organisms occurred in the joinpoint period from H1 of 2012 to H2 of 2014. After excluding MRSA, similar results were observed. In both the five-hospital and seven-hospital analyses, there were significant decreases in utilisation rate of third-generation cephalosporins and in one of the joinpoint periods for carbapenems whereas utilisation rates of IV amoxicillin-clavulanate and piperacillin-tazobactam increased significantly. In the five-hospital analysis, utilisation rates of anti-pseudomonal antibiotics and quinolones decreased significantly. In the last joinpoint periods, half-yearly incidence density of ceftriaxone-resistant *E. coli* and *K. pneumoniae*, MDR and imipenem or meropenem-resistant *A. baumannii*, MRSA and *C. difficile* decreased significantly while ciprofloxacin-resistant *E. coli* and *K. pneumoniae* increased significantly. Overall, imipenem or meropenem-resistant *E. coli* and *K. pneumoniae* increased significantly.

There were no significant changes in overall broad-spectrum antibiotic utilisation rate in the seven-hospital analysis in contrast to the analysis in the five-hospital analysis. The data for the seven-hospital analysis included a specialist women’s and children’s hospital which could have resulted in overall lower antibiotic utilisation rate because of the high proportion of paediatric doses and higher prevalence of generally well, young and healthy women. Findings from the five-hospital analysis suggested that with systematic implementation of nationally funded resource-intensive ASP activities based on evidence and local antibiograms, broad-spectrum antibiotic utilisation rate declined during the study period [2–5].

Although most of the hospitals provided PRF on carbapenem and piperacillin-tazobactam prescriptions and had a high acceptance rate of ASP interventions, we were unable to contain the rise in piperacillin-tazobactam usage. However, the increase could have been more substantial without ASP. The increase in utilisation rate of levofloxacin during the study period could be attributed to its increased use in the national tuberculosis treatment unit of one hospital (data unpublished).

Institutional guidelines encouraged the empiric use of antibiotics based on local hospital antibiogram sand international guidelines while expert practice usually relies on a few “work-horse” antibiotics which could explain the high initial utilisation rate of ceftriaxone and PO ciprofloxacin^5^. PRF provided patient and prescriber level stewardship while computerised decision support systems and educational campaigns increased awareness of institutional antibiotic guidelines and built the culture of judicious antibiotic use. Annual reports were disseminated by NARCC to the respective hospital management based on data submitted as a form of benchmarking. This not only validated the national antibiotic stewardship programme but helped to engage hospital leadership for continuing support. Although we were unable to attribute these activities to the changes in antibiotic utilisation in our study, these activities could have contributed to the trends observed [6–11, 19].

Antibiotic use creates a selective pressure towards the emergence of antibiotic resistance. In the five-hospital analysis based on half-yearly interval, the decrease in utilisation rate of broad-spectrum antibiotics preceded the decrease in incidence density of antibiotic-resistant organisms. The decrease in utilisation of ceftriaxone preceded the decrease in incidence density of ceftriaxone-resistant *E. coli* and *K. pneumoniae*. It was previously reported that there was a positive association between increased use of ceftriaxone and the increase in extended-spectrum beta-lactamases that confer ceftriaxone resistance [20]. The decrease in utilisation of quinolones and anti-pseudomonal antibiotics occurred before the decrease in incidence density of *C. difficile*. In a local study, utilisation rate of broad-spectrum antibiotics had a significant negative correlation with incidence density of *C. difficile* and carbapenem-resistant *P. aeruginosa* but not with carbapenem-resistant *A. baumannii* [21]. The significant decreases in incidence density of MRSA and carbapenem-resistant and MDR *A*. *baumannii* in our current study could be due to infection control practices but ASP remains important in controlling the incidence of these MDRO, including *C. difficile* [22–24]. The presence of community-acquired extended spectrum beta-lactamase -producing *E. coli* and *K. pneumoniae* locally could have affected the overall incidence density of ceftriaxone-resistant *E. coli* and *K. pneumoniae* [25–27]. The increase in carbapenem-resistant *Enterobacterales* locally was previously reported [28].

There are a few limitations in our study. As the reporting intervals of antibiotic utilisation and incidence density of antibiotic-resistant organisms to NARCC were different, we converted the quarterly time series of antibiotic utilisation rate to half-yearly intervals for comparison. We were unable to report on all antibiotics and hence may not be able to address “balloon effects” adequately. Nonetheless, we reported on antibiotics that were commonly regarded as broad-spectrum and those from the WHO watch list as they were regarded as targets of ASP monitoring [29]. We could not determine if the antibiotic-resistant organisms reported were community-acquired or healthcare-associated. The possible impact of infection control measures and other hospital activities were unaccounted for.

The work to ensure judicious use of antibiotics at our local hospitals where there is high antibiotic resistance is ongoing [30]. The first decade of ASP work in Singapore is encouraging but it is not always the case from overseas experience [31]. Reports on reduction in ASP activities due to the COVID-19 pandemic highlights the importance of sustaining ASP to reduce the overuse of antibiotics and antibiotic resistance in the long-run [32, 33].

## Conclusions

Our study demonstrated reductions in the use of several types of antibiotics after nationally funded AMR surveillance and ASP were implemented, which were followed by reductions in antimicrobial resistance across several classes. Although there were increases in some classes of antimicrobial resistance and utilisation of some types of antibiotics, these findings suggest that ASP should continue to be funded nationally as a key measure to combat antimicrobial resistance in acute care hospitals.

### Electronic supplementary material

Below is the link to the electronic supplementary material.

Supplementary Material 1

## Data Availability

The data that supports this research are available from the corresponding author upon reasonable request.
