# Supplementary material for: Antibiotic utilisation and resistance over the first decade of nationally funded antimicrobial stewardship programmes in Singapore acute-care hospitals
Source: Antimicrob Resist Infect Control. 2023 Aug 23;12:82. doi: 10.1186/s13756-023-01289-x (PMC10464409; doi:10.1186/s13756-023-01289-x)
Supplement: Supplementary file 1 — Supplementary Material 1 [file 13756_2023_1289_MOESM1_ESM.docx]

**Supplementary materials**

**Table S1. Joinpoint regression analysis of broad-spectrum antibiotic utilisation rate (defined daily doses per 1,000 inpatient-days) across seven public acute-care hospitals, quarter 1 of 2011 to quarter 4 of 2020**

| **Antibiotic** | **DDDs/1,000 inpatient-days** | | **Total study period (Q1 2011–Q4 2020)** | **Trend 1** | | **Trend 2** | | **Trend 3** | | **Trend 4** | | **Trend 5** | |
| --- | --- | --- | --- | --- | --- | --- | --- | --- | --- | --- | --- | --- | --- |
|  | **Q1 2011** | **Q4 2020** | **AQPC (%), (95% CI)** | **Quarters** | **QPC (%),**  **(95% CI)** | **Quarters** | **QPC (%), (95% CI)** | **Quarters** | **QPC (%), (95% CI)** | **Quarters** | **QPC (%), (95% CI)** | **Quarters** | **QPC (%), (95% CI)** |
| **All^1^** | 268.6 | 261.0 | -0.1  (-0.2, 0.01) |  |  |  |  |  |  |  |  |  |  |
| **Third generation cephalosporins^2^** | 97.8 | 65.5 | -1.1**  (-1.8, -0.3) | Q1 2011–Q1 2013 | -2.0***  (-2.8, -1.1) | Q1 2013–Q4 2013 | -7.6  (-15.9, 1.6) | Q4 2013–Q3 2015 | 1.3  (-0.05, 2.7) | Q3 2015–Q3 2017 | -1.6**  (-2.6, -0.5) | Q3 2017–Q4 2020 | 0.1  (-0.3, 0.5) |
| Ceftazidime, IV (g) | 8.9 | 8.6 | 0.1  (-0.9, 1.1) | Q1 2011–Q4 2011 | 8.2  (-2.3, 19.8) | Q4 2011–Q3 2013 | -4.1*  (-7.1, -1.0) | Q3 2013–Q4 2020 | 0.3*  (0.04, 0.6) |  |  |  |  |
| Ceftriaxone, IV (g) | 89.0 | 56.8 | -1.2**  ( -2.0, -0.3) | Q1 2011–  Q1 2013 | -2.3***  (-3.0, -1.1) | Q1 2013–Q4 2013 | -7.6  (-16.4, 2.0) | Q4 2013–Q3 2015 | 1.1  (-0.4, 2.7) | Q3 2015–Q4 2017 | -1.5**  (-2.4, -0.5) | Q4 2017–Q4 2020 | 0.1  (-0.5, 0.6) |
| **Carbapenems^3^** | 42.1 | 34.2 | -0.7***  (-1.1, -0.3) | Q1 2011–Q1 2016 | 0.02  (-0.5, 0.5) | Q1 2016–Q4 2020 | -1.4***  (-2.0, -0.8) |  |  |  |  |  |  |
| Doripenem, IV (g) | 0.3 | 0.2 | -2.7*  (-5.2, -0.1) |  |  |  |  |  |  |  |  |  |  |
| Ertapenem, IV (g) | 9.8 | 7.7 | -0.5  (-1.2, 0.3) | Q1 2011–Q1 2012 | 2.9  (-3.4, 9.6) | Q1 2012–Q1 2016 | -1.6***  (-2.4, -0.9) | Q1 2016–Q4 2020 | -0.2  (-0.7, 0.3) |  |  |  |  |
| Imipenem, IV (g) | 8.8 | 0.8 | -6.8***  (-7.7, -5.9) | Q1 2011–Q2 2016 | -9.3***  (-10.4, -8.2) | Q2 2016–Q4 2020 | -3.7***  (-5.2, -2.2) | - |  |  |  |  |  |
| Meropenem, IV (g) | 23.2 | 25.6 | -0.0004  (-0.8, 0.7) | Q1 2011–Q4 2013 | 0.8  (-0.5, 2.2) | Q4 2013–Q3 2015 | 3.8*  (0.1, 7.6) | Q3 2015–Q4 2020 | -1.7***  (-2.2, -1.2) |  |  |  |  |
| **Quinolones^4^** |  |  |  |  |  |  |  |  |  |  |  |  |  |
| Ciprofloxacin, IV (g) | 8.0 | 2.8 | -2.7*  (-4.8, -0.6) | Q1 2011–Q3 2016 | -2.0***  (-2.4, -1.6) | Q3 2016–Q3 2018 | 0.3  (-2.1,2.8) | Q3 2018–Q2 2019 | -15.3  (-35.8, 11.7) | Q2 2019–Q4 2020 | -2.8  (-5.9, 0.3) |  |  |
| **Beta-lactam/beta-lactamase inhibitors^5^** | 78,8 | 122.2 | 1.3***  (1.1-1.5) | Q1 2011–  Q2 2013 | 2.3***  (1.4, 3.2) | Q2-2013–Q4 2020 | 1.0***  (0.8, 1.1) |  |  |  |  |  |  |
| Amoxicillin-clavulanate, IV (g) | 40.1 | 62.0 | 1.5***  (1.3, 1.6) |  |  |  |  |  |  |  |  |  |  |
| Piperacillin-tazobactam, IV (g) | 38.7 | 60.2 | 1.2***  (0.9, 1.5) | Q1 2011–Q1 2013 | 3.8***  (2.4, 5.2) | Q1 2013–Q4 2020 | 0.5***  (0.3, 0.7) |  |  |  |  |  |  |
| **Others** |  |  |  |  |  |  |  |  |  |  |  |  |  |
| Cefepime, IV (g) | 11.5 | 7.4 | -1.2  (-3.5, 1.1) | Q1 2011–Q3 2011 | 22.8  (-6.1, 60.7) | Q3 2011–Q3 2013 | -4.0**  (-6.1, -2.0) | Q3 2013–Q3 2018 | 0.7**  (0.2, 1.1) | Q3 2018–Q2 2019 | -19.4  (-36.9, 3.1) | Q2 2019-Q4 2020 | -0.9  (-3.6, 2.0) |
| Colistin, IV (million units) | 0.5 | 0.03 | -6.0***  (-8.5, -3.5) | Q1 2011–Q3 2015 | 0.3  (-3.9, 4.7) | Q3 2015–Q4 2020 | -11.2***  (-14.1, -8.1) |  |  |  |  |  |  |
| Polymyxin B, IV (g) | 1.4 | 0.3 | -4.9***  (-6.7, -3.0) | Q1 2011–Q2 2013 | 6.3*  (1.1, 11.8) | Q2 2013–Q3 2019 | -5.3***  (-6.4, -4.2) | Q3 2019–Q4 2020 | -20.2***  (-29.2, -10.2) |  |  |  |  |
| Tigecycline, IV (g) | 0.8 | 1.0 | 0.4  (-0.5, 1.3) |  |  |  |  |  |  |  |  |  |  |
| Linezolid, IV and PO (g) | 2.6 | 2.5 | 0.2  (-1.1, 1.5) | Q1 2011–Q2 2018 | -4.0***  (-4.9, -3.2) | Q2 2018–Q4 2020 | 13.5***  (8.4, 18.8) |  |  |  |  |  |  |
| Daptomycin, IV (g) | 1.1 | 2.5 | 2.7  (-2.6, 8.3) | Q1 2011–Q3 2011 | -26.1  (-55.3, 22.1) | Q3 2011–Q3 2012 | 32.0*  (4.5, 66.7) | Q3 2012–Q4 2013 | -9.9  (-21.9, 4.0) | Q4 2013–Q3 2014 | 27.9  (-22.4, 110.8) | Q3 2014–Q4 2020 | 1.3**  (0.5, 2.2) |
| Vancomycin IV (g) | 24.1 | 22.6 | -0.1  (-0.8, 0.5) | Q1 2011–Q4 2011 | 3.9  (-1.9, 10.1) | Q4 2011–Q4 2013 | -3.3***  (-4.4, -2.1) | Q4 2013–Q4 2015 | 2.0**  (0.8, 3.3) | Q4 2015–Q2 2017 | -2.3*  ( -4.3, -0.2) | Q2 2017–Q4 2020 | 0.5*  (0.1, 0.9) |

* *p*<0.05; ** *p*<0.01; ****p* < 0.001.

CI: confidence interval, DDDs: daily defined doses, Q: quarter, QPC: quarterly percentage change, AQPC: average quarterly percentage change, IV: intravenous, PO: oral.

^1^Excluded ciprofloxacin PO, levofloxacin IV and PO, and moxifloxacin IV and PO. ^2^Third generation cephalosporins comprise ceftazidime and ceftriaxone. ^3^Carbapenems comprise ertapenem, imipenem, meropenem and doripenem. ^4^Quinolones comprise ciprofloxacin, levofloxacin, and moxifloxacin. ^5^Beta-lactam/beta-lactamase inhibitors comprise amoxicillin-clavulanate and piperacillin-tazobactam.

**Table S2. Joinpoint regression analysis of antibiotic utilisation rate (defined daily doses per 1,000 inpatient-days) across five public acute care hospitals, quarter1 of 2011 to quarter 4 of 2020**

| **Antibiotic** | **DDDs/1,000 inpatient-days** | | **Total study period (Q1 2011–Q4 2020)** | **Trend 1** | | **Trend 2** | | **Trend 3** | | **Trend 4** | | **Trend 5** | | **Trend 6** | |
| --- | --- | --- | --- | --- | --- | --- | --- | --- | --- | --- | --- | --- | --- | --- | --- |
|  | **Q1 2011** | **Q4 2020** | **AQPC (%) (95% CI)** | **Quarters** | **QPC (%)**  **(95% CI)** | **Quarters** | **QPC (%)**  **(95% CI)** | **Quarters** | **QPC (%)**  **(95% CI)** | **Quarters** | **QPC (%)**  **(95% CI)** | **Quarters** | **QPC (%)**  **(95% CI)** | **Quarters** | **QPC (%)**  **(95% CI)** |
| **All** | 714.6 | 509.6 | -0.8**  (-1.3, -0.4) | Q1 2011–Q4 2012 | -0.7  (-1.8, 0.5) | Q4 2012–Q4 2013 | -4.7*  (-8.6, -0.5) | Q4 2013–Q4 2020 | -0.3***  (-0.5, -0.2) |  |  |  |  |  |  |
| **Anti-pseudomonal antibiotics^1^** | 526.4 | 353.5 | -1.0***  (-1.5, -0.5) | Q1 2011–Q4 2012 | -0.6  (-1.9, 0.7) | Q4 2012–Q4 2013 | -5.3*  (-9.7, -0.7) | Q4 2013–Q4 2020 | -0.5***  (-0.6, -0.3) |  |  |  |  |  |  |
| **Third generation cephalosporins^2^** | 101.8 | 61.1 | -1.3**  (-2, -0.5) | Q1 2011–Q1 2013 | -1.8***  (-2.5, -1.1) | Q1 2013–Q4 2013 | -7.3  (-15.4, 1.6) | Q4 2013–Q2 2015 | 0.8  (-0.8, 2.4) | Q2 2015–Q3 2019 | -1.2***  (-1.4. -0.9) | Q3 2019–Q4 2020 | 0.6  (-1.0, 2.3) |  |  |
| Ceftazidime, IV (g) | 8.6 | 8.3 | 0.2  (-1.5, 1.8) | Q1–Q4 2011 | 11.9*  (2.1, 22.6) | Q4 2011–Q3 2012 | -7.1  (-24.3, 14.0) | Q3 2012–Q4 2020 | -0.2  (-0.4, 0.04) |  |  |  |  |  |  |
| Ceftriaxone, IV (g) | 93.1 | 52.8 | -1.4***  (-2.2, -0.6) | Q1 2011–Q1 2013 | -2.0**  (-2.7, -1.2) | Q1 2013–Q4 2013 | -7.8  (-16.2, 1.5) | Q4 2013–Q2 2015 | 0.7  (-0.9, 2.4) | Q2 2015–Q3 2019 | -1.3***  (-1.5. -1.0) | Q3 2019–Q4 2020 | 0.6  (-1.0, 2.3) |  |  |
| **Carbapenems^3^** | 47.3 | 37.3 | -0.7  (-2.2, 0.8) | Q1 2011–Q3 2018 | -0.2*  (-0.4, -0.02) | Q3 2018–Q2 2019 | -7.9  ( -24.5, 12.5) | Q2 2019–Q4 2020 | 0.7  (-1.8, 3.4) |  |  |  |  |  |  |
| Doripenem, IV (g) | 0.4 | 0.2 | -3.1*  (-5.7, -0.5) |  |  |  |  |  |  |  |  |  |  |  |  |
| Ertapenem, IV (g) | 11.4 | 8.9 | -0.7***  (-0.9, -0.5) |  |  |  |  |  |  |  |  |  |  |  |  |
| Imipenem, IV (g) | 9.8 | 0.8 | -6.3***  (-7.9, -4.8) | Q1 2011–Q2 2012 | -3.2  (-13.1, 7.9) | Q2 2012–Q2 2016 | -10.7***  (-12.4, -9.0) | Q2 2016–Q4 2020 | -3.2***  (-4.5, -1.8) |  |  |  |  |  |  |
| Meropenem, IV (g) | 25.8 | 27.3 | 0.1  (-1.8, 2.0) | Q1 2011–Q4 2015 | 1.8***  (1.3, 2.3) | Q4 2015–Q3 2018 | -0.7  (-2.0, 0.7) | Q3 2018–Q2 2019 | -8.7  (-28.4, 16.3) | Q2 2019–Q4 2020 | 0.9  (-2.2, 4.1) |  |  |  |  |
| **Quinolones^4^** | 431.3 | 247.5 | -1.4**  (-2.3, -0.5) | Q1 2011–Q1 2013 | -1.6*  (-3.0, -0.3) | Q1 2013–Q4 2013 | -9.3  (-19, 1.5) | Q4 2013–Q4 2020 | -0.5***  (-0.7, -0.2) |  |  |  |  |  |  |
| Ciprofloxacin, IV (g) | 8.3 | 2.9 | -2.6**  (-4.2, -0.9) | Q1 2011–Q1 2013 | -0.2  (-1.7, 1.4) | Q1 2013–Q2 2015 | -3.4, -4.8- -1.9*** | Q2 2015–Q3 2018 | -0.1  (-0.9, 0.7) | Q3 2018–Q2 2019 | -15.6  (-31.6, 4.1) | Q2 2019–Q4 2020 | -2.8*  (-5.1, -0.4) |  |  |
| Ciprofloxacin, PO (g) | 372.8 | 165.4 | -2.0***  (-2.9, -1.2) | Q1 2011–Q1 2013 | -2.5***  (-3.8, -1.3) | Q1 2013–Q4 2013 | -8.2  (-18.1, 2.8) | Q1 2013–Q4 2020 | -1.2***  (-1.4, -1.0) |  |  |  |  |  |  |
| Levofloxacin, IV and PO (g) | 41.9 | 78.0 | 1.5  (-0.1, 3.2) | Q1 2011–Q4 2012 | 3.5**  (1.2, 5.9) | Q4 2012–Q2 2015 | -3.1***  (-4.6, -1.6) | Q2 2015–Q1 2016 | 13.4  (-3.8, 33.8) | Q1 2016– Q2 2017 | -5.2*  (-10.2, -0.03) | Q2 2017 –Q3 2019 | 6.8***  (4.8, 8.8) | Q3 2019 –Q4 2020 | -0.7  (-4.4, 3.2) |
| Moxifloxacin, IV and PO (g) | 8.4 | 1.2 | -4.9*  (-8.9, -0.7) | Q1 2011–Q3 2017 | -5.9***  (-6.8, -5.0) | Q3 2017–Q2 2018 | 17.4  (-33.0, 105.7) | Q2 2018–Q4 2020 | -8.1***  (-11.7, -4.3) |  |  |  |  |  |  |
| **Beta-lactam/beta-lactamase inhibitors^5^** | 86.5 | 124.6 | 0.9  (0.0, 1.8) | Q1 2011–Q2 2014 | 1.9***  (1.6, 2.3) | Q2 2014– Q4 2016 | 0.2  (-0.4, 0.8) | Q4 2016– Q3 2018 | 1.7**  (0.6, 2.9) | Q3 2018– Q3 2019 | -0.9  (-4.5, 2.9) | Q3 2019– Q2 2020 | 3.5  (-5.0, 12.8) | Q2 2020 –Q4 2020 | -5.6  (-13.6, 3.0) |
| Amoxicillin-clavulanate, IV (g) | 43.0 | 62.1 | 1.0**  (0.4, 1.7) | Q1 2011–Q4 2012 | -0.02  (-1.4, 1.3) | Q4 2012–Q1 2014 | 3.5*  (0.1, 6.9) | Q1 2014–Q4 2015 | -0.3  (-2, 1.4) | Q4 2015–Q3 2018 | 2.4***  (1.6, 3.2) | Q3 2018–Q4 2020 | -0.1  (-1, 0.8) |  |  |
| Piperacillin-tazobactam, IV (g) | 43.5 | 62.5 | 1.0***  (0.7, 1.3) | Q1 2011–Q1 2013 | 3.7***  (2.6, 4.9) | Q1 2013–Q4 2020 | 0.3***  (0.2, 0.4) |  |  |  |  |  |  |  |  |
| **Others** |  |  |  |  |  |  |  |  |  |  |  |  |  |  |  |
| Cefepime, IV (g) | 13.2 | 7.7 | -1.5  (-3.6, 0.7) | Q1 2011-Q3 2011 | 22.3  (-1.0, 0.8) | Q3 2011-Q3 2013 | -4.0**  (-6.2, -1.8) | Q3 2013-Q3 2018 | 0.6*  (0.1, 1.1) | Q3 2018-Q2 2019 | -19.3  (-36.4, 2.3) | Q2 2019- Q4 2020 | -2.1  (-5.0, 0.9) |  |  |
| Colistin, IV (million units) | 0.5 | 0.04 | -4.5**  (-7.5, -1.3) | Q1 2011–Q2 2013 | 11.0  (-2.5, 26.4) | Q2 2013–Q4 2020 | -8.7***  (-10.5, -6.8) |  |  |  |  |  |  |  |  |
| Polymyxin B, IV (g) | 1.6 | 0.3 | -4.8***  (-6.6, -2.9) | Q1 2011–Q2 2013 | 6.1*  (1.1, 11.4) | Q2 2013–Q3 2019 | -5.0***  (-6.1, -4.0) | Q3 2019–Q4 2020 | -20.6***  (-29.3, -10.8) |  |  |  |  |  |  |
| Tigecycline, IV (g) | 0.9 | 1.2 | 0.5  (-0.3, 1.4) |  |  |  |  |  |  |  |  |  |  |  |  |
| Linezolid, IV and PO (g) | 3.0 | 2.6 | 0.0002  (-1.2, 1.3) | Q1 2011–Q2 2018 | -3.9***  (-4.8, -3.1) | Q2 2018–Q4 2020 | 12.4***  (7.6, 17.5) |  |  |  |  |  |  |  |  |
| Daptomycin, IV (g) | 1.2 | 2.9 | 2.8  (-2.6, 8.4) | Q1–Q3 2011 | -26.4  (-55.6, 21.9) | Q3 2011–Q3 2012 | 32.2*  (3.9, 68.3) | Q3 2012–Q4 2013 | -9.9  (-22.4, 4.6) | Q4 2013–Q3 2014 | 28.0  (-22.7, 111.8) | Q3 2014–Q4 2020 | 1.4**  (0.5, 2.3) |  |  |
| Vancomycin IV (g) | 27.2 | 24.3 | -0.2  (-1.3, 0.9) | Q1–Q4 2011 | 4.4, -1.1-10.1 | Q4 2011–Q3 2013 | -3.6***  (-5.0, -2.2) | Q3 2013–Q3 2016 | 1.2***  (0.7, 1.8) | Q3 2016–Q2 2017 | -4.1  (-16.1, 9.5) | Q2 2017–Q4 2020 | 0.2  (-0.2, 0.6) |  |  |

* *p*<0.05; ** *p*<0.01; ****p* < 0.001.

CI: confidence interval, DDDs: daily defined doses, Q: quarter, QPC: quarterly percentage change, AQPC: average quarterly percentage change, IV: intravenous, PO: oral.

^1^Anti-pseudomonal antibiotics comprise cefepime, ceftazidime, ciprofloxacin, colistin, doripenem, imipenem, meropenem, levofloxacin, piperacillin-tazobactam and polymyxin B. ^2^Third generation cephalosporins comprise ceftazidime and ceftriaxone. ^3^Carbapenems comprise ertapenem, imipenem, meropenem and doripenem. ^4^Quinolones comprise ciprofloxacin, levofloxacin, and moxifloxacin. ^5^Beta-lactam/beta-lactamase inhibitors comprise amoxicillin-clavulanate and piperacillin-tazobactam.


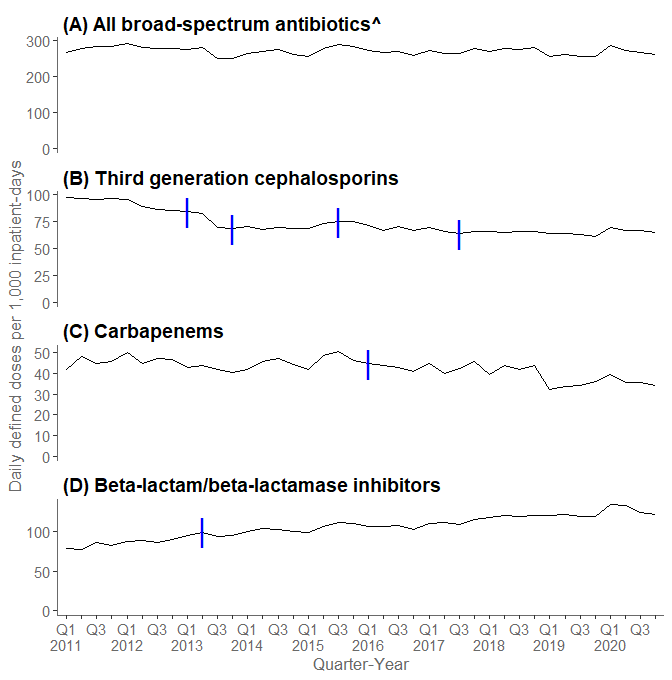


**Figure S1.** Trends in antibiotic utilisation rate (daily defined doses per 1,000 inpatient-days) across seven public acute-care hospitals for (A) All broad-spectrum antibiotics^1^, (B) Third-generation cephalosporins, (C) Carbapenems, (D) Beta-lactam/beta-lactamase inhibitors, quarter 1 of 2011 to quarter 4 of 2020. The | symbols denote the joinpoints identified using joinpoint regression analysis.

^1^Excluded ciprofloxacin PO, levofloxacin IV and PO, and moxifloxacin IV and PO.

Third generation cephalosporins comprise ceftazidime and ceftriaxone. Carbapenems comprise ertapenem, imipenem, meropenem, and doripenem. Beta-lactam/beta-lactamase inhibitors comprise amoxicillin-clavulanate and piperacillin-tazobactam.


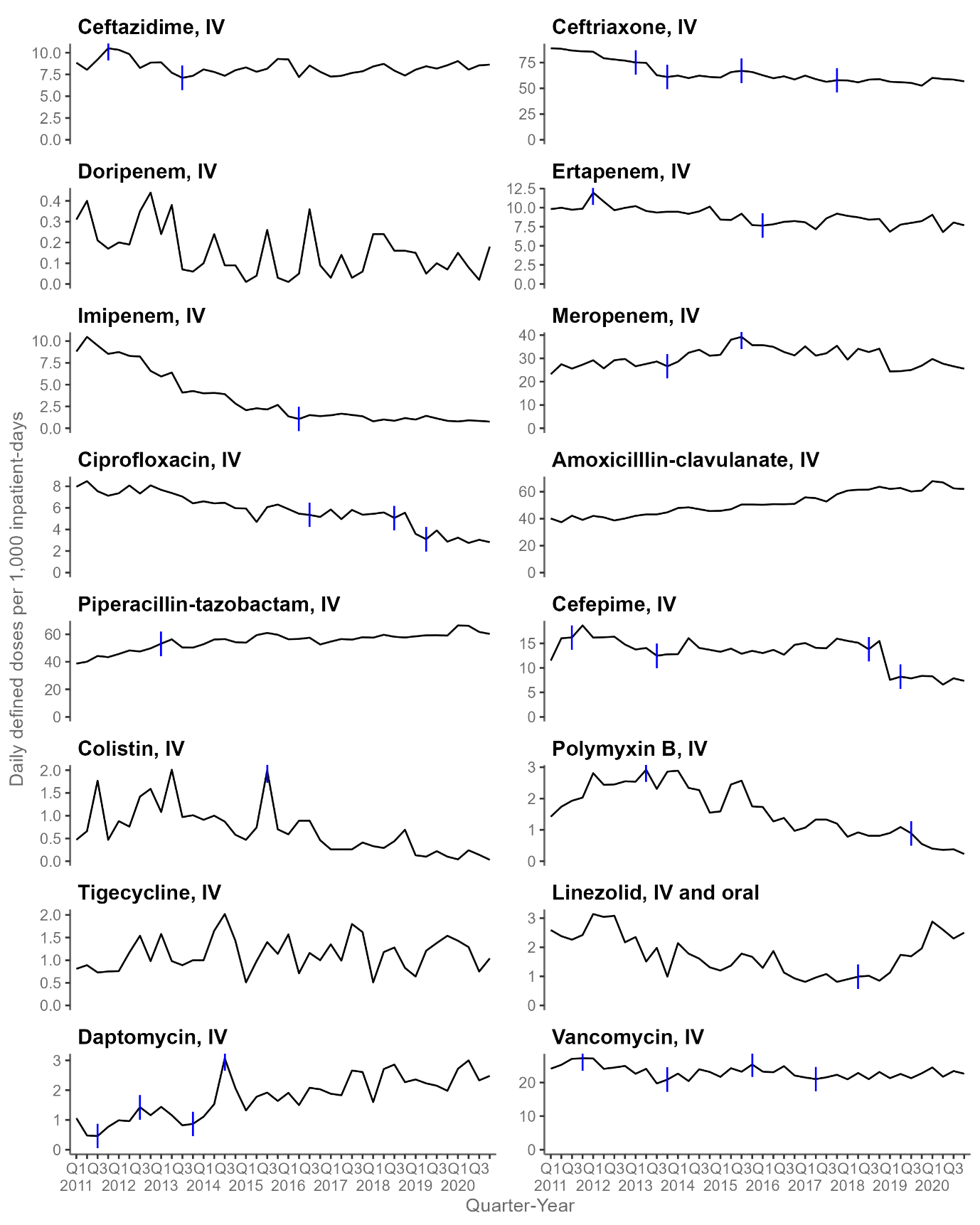
**Figure S2.** Trends in antibiotic utilisation rate (daily defined doses per 1,000 inpatient-days) across seven public acute-care hospitals, quarter 1 of 2011 to quarter 4 of 2020. The | symbols denote the joinpoints identified using joinpoint regression analysis.


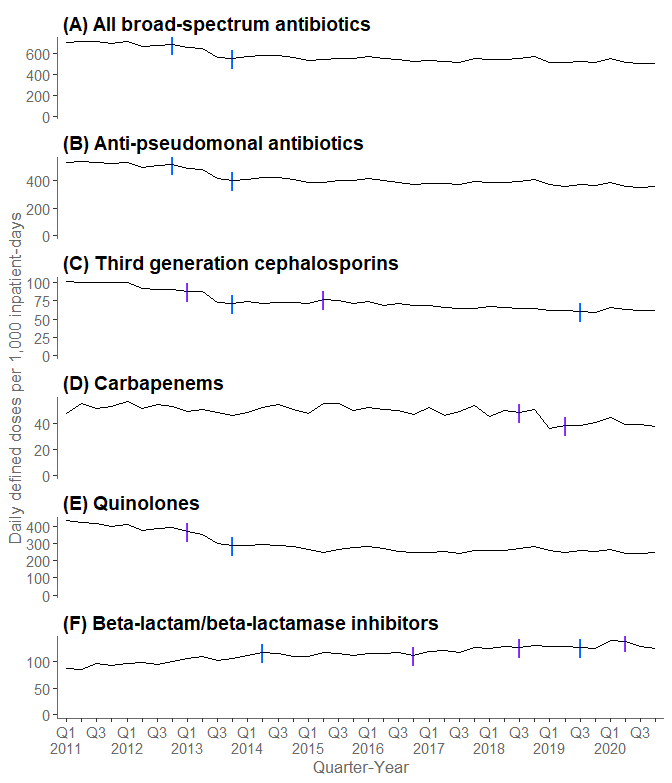


**Figure S3.** Trends in antibiotic utilisation rate (daily defined doses per 1,000 inpatient-days) across five public acute-care hospitals for (A) All broad-spectrum antibiotics, (B) Anti-pseudomonal antibiotics, (C) Third-generation cephalosporins, (D) Carbapenems, (E) Quinolones, (F) Beta-lactam/beta-lactamase inhibitors, quarter 1 of 2011 to quarter 4 of 2020. The | symbols denote the joinpoints identified using joinpoint regression analysis.

Anti-pseudomonal antibiotics comprise cefepime, ceftazidime, ciprofloxacin, colistin, doripenem, imipenem, meropenem, levofloxacin, piperacillin-tazobactam and polymyxin B. Third generation cephalosporins comprise ceftazidime and ceftriaxone. Carbapenems comprise ertapenem, imipenem, meropenem and doripenem. Quinolones comprise ciprofloxacin, levofloxacin and moxifloxacin. Beta-lactam/beta-lactamase inhibitors comprise amoxicillin-clavulanate and piperacillin-tazobactam.


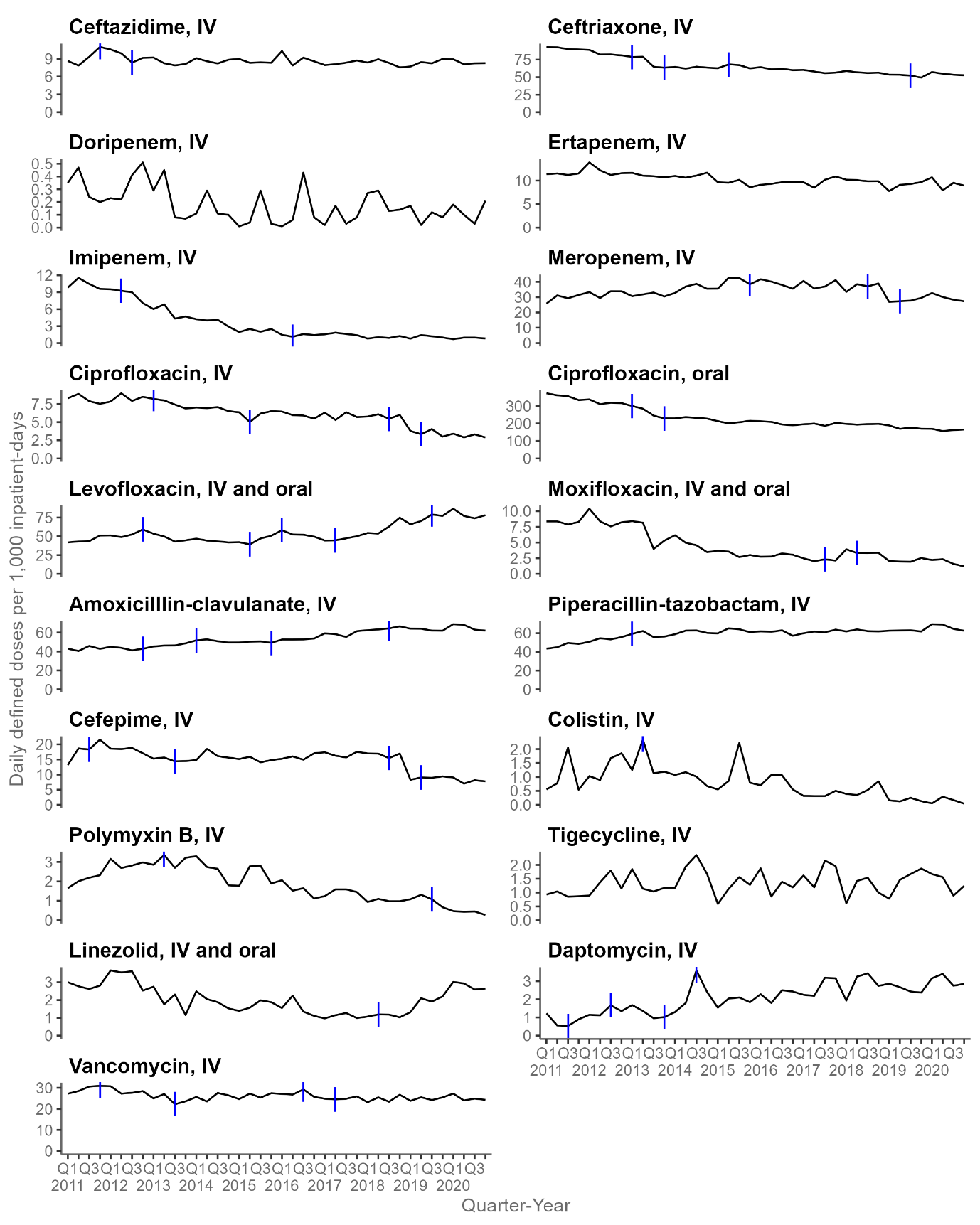


**Figure S4.** Trends in antibiotic utilisation (daily defined doses per 1,000 inpatient-days) across five public acute-care hospitals, quarter 1 of 2011 to quarter 4 of 2020. The | symbols denote the joinpoints identified using joinpoint regression analysis.


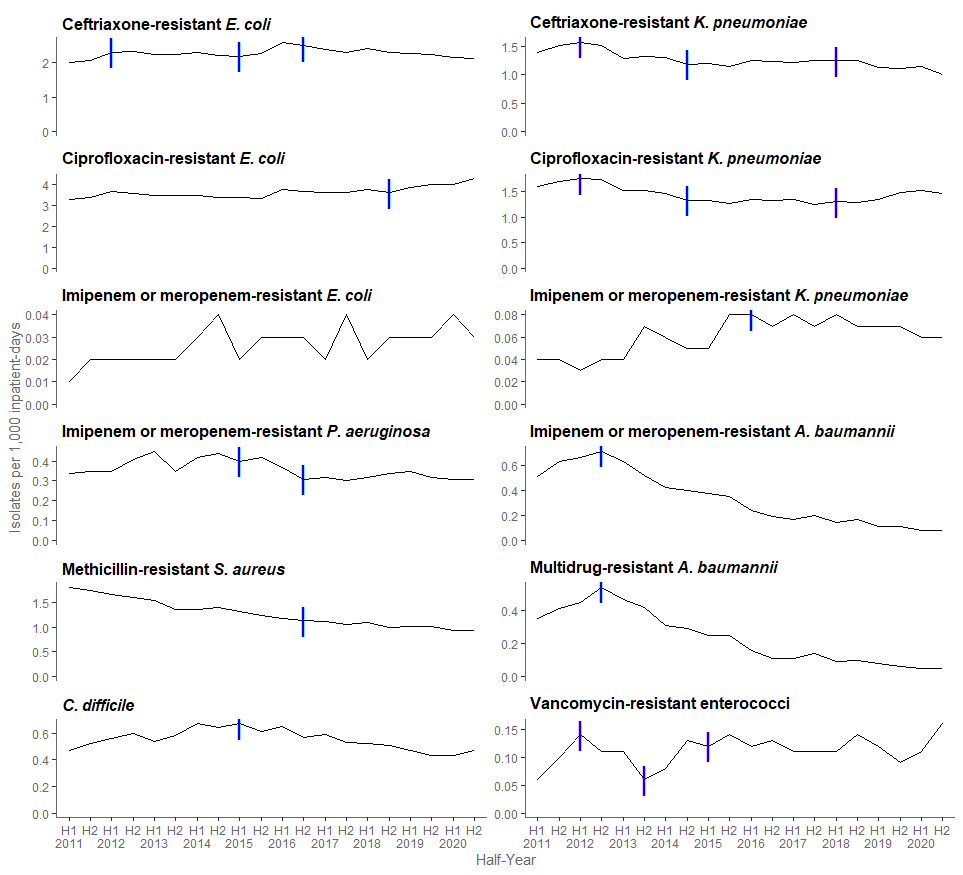


**Figure S5.** Trends in incidence density of antibiotic resistant organisms (isolates per 1,000 inpatient-days) across seven public acute-care hospitals, first half of 2011 to second half of 2020. The | symbols denote the joinpoints identified using joinpoint regression analysis.


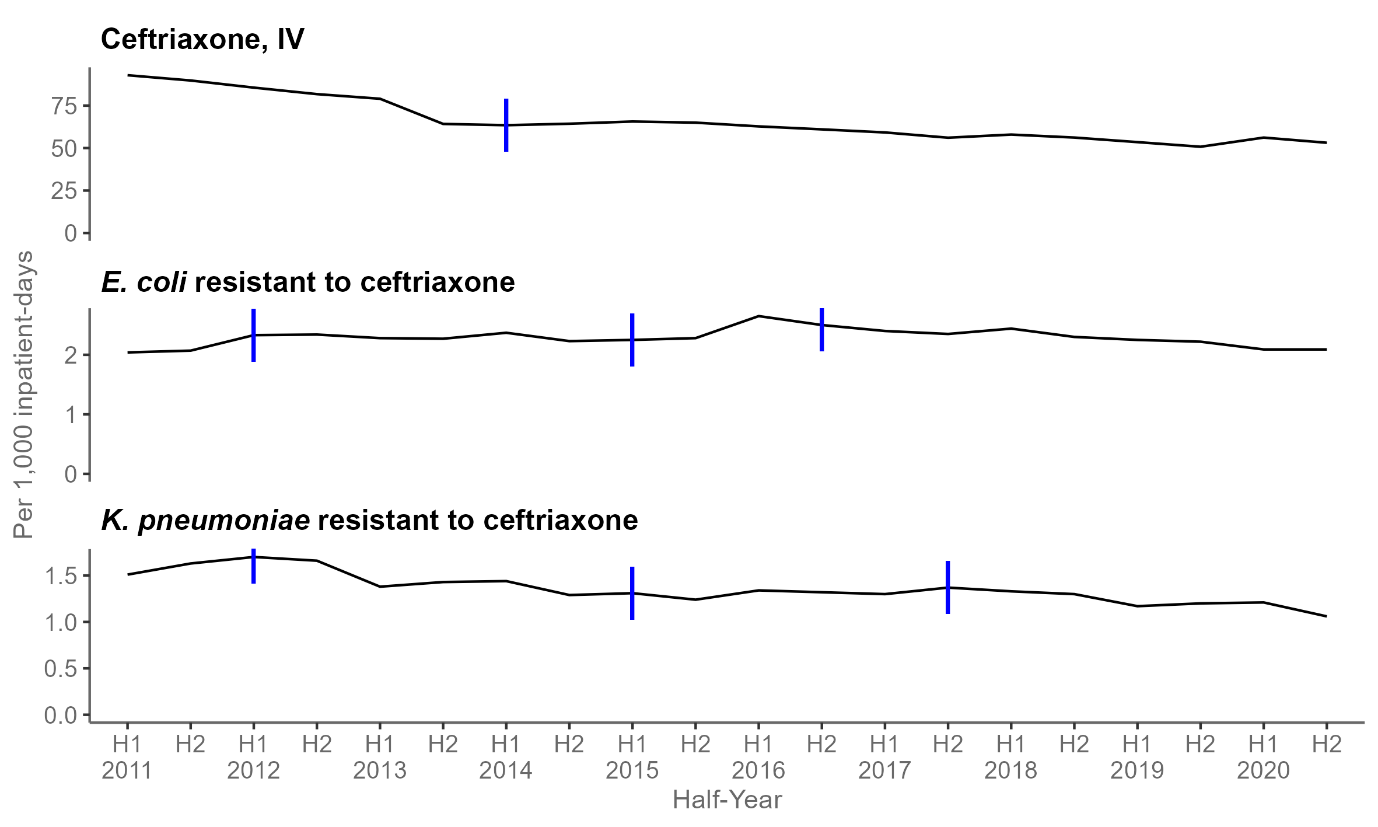


**Figure S6.** Trends in antibiotic utilisation rate (daily defined doses per 1,000 inpatient-days) of ceftriaxone and incidence density of ceftriaxone-resistant *E. coli* and *K. pneumoniae* (isolates per 1,000 inpatient-days) across five public acute-care hospitals, first half of 2011 to second half of 2020. The | symbols denote the joinpoints identified using joinpoint regression analysis.


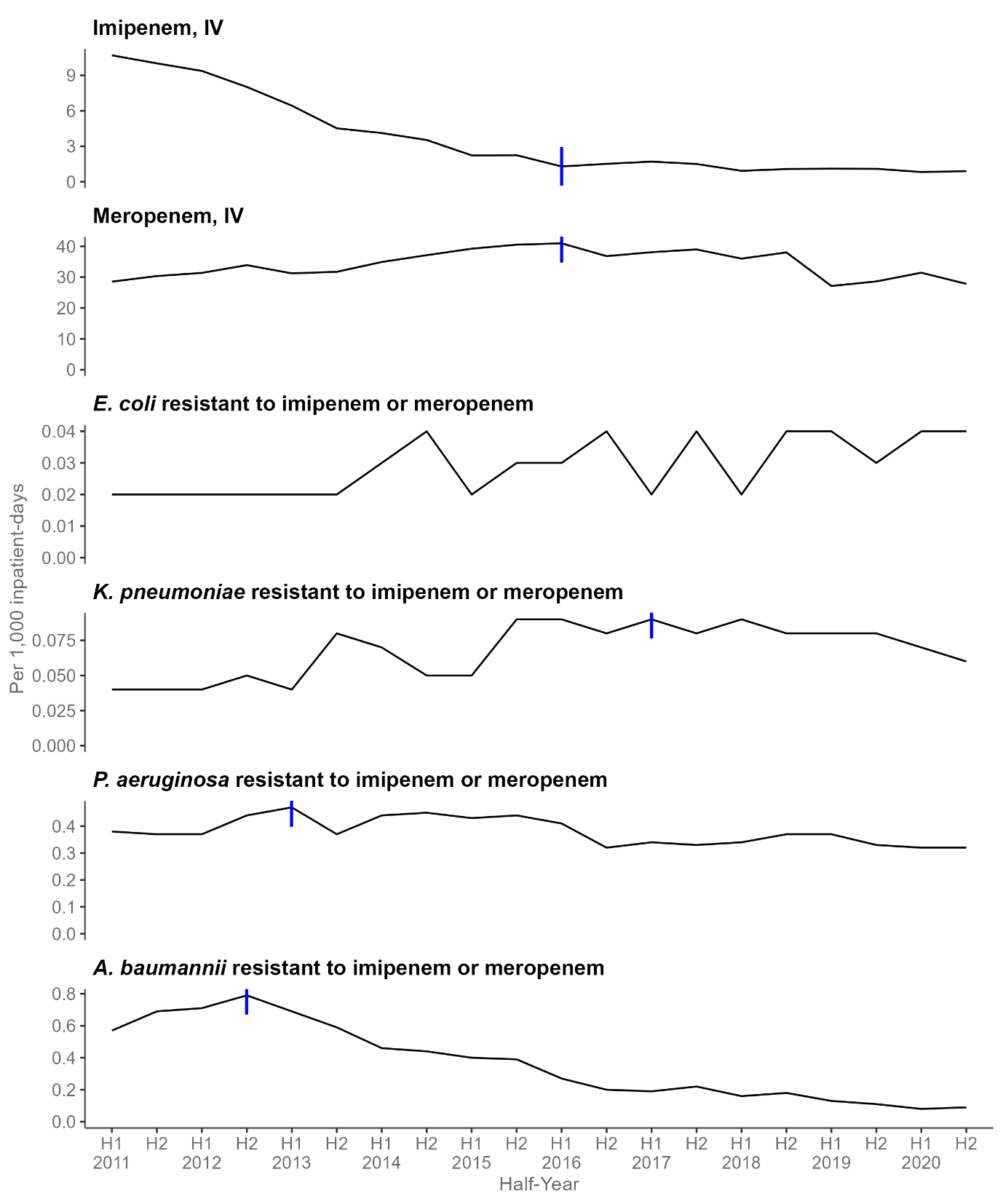
**Figure S7.** Trends in antibiotic utilisation rate (daily defined doses per 1,000 inpatient-days) of imipenem and meropenem, and incidence density of imipenem or meropenem-resistant *E. coli, K. pneumoniae, P. aeruginosa* and *A. baumannii* (isolates per 1,000 inpatient-days) across five public acute-care hospitals, first half of 2011 to second half of 2020. The | symbols denote the joinpoints identified using joinpoint regression analysis.
